# The Addition of an Acid Protease Improved the Digestibility of Crude Protein and Amino Acids of Soybean Meal, but Not of Corn, in Piglets

**DOI:** 10.3390/ani15203037

**Published:** 2025-10-20

**Authors:** Leury J. Souza, José A. L. Barbosa, Hélio Moreira Júnior, Francisco A. Pereira, Marcos L. P. Tse, Urbano S. Ruiz

**Affiliations:** 1 Department of Animal Science, “Luiz de Queiroz” College of Agriculture, University of São Paulo, Piracicaba 13418-900, SP, Brazil; leuryjsouza@gmail.com (L.J.S.);; 2 Department of Animal Nutrition and Production, School of Veterinary Medicine and Animal Science, University of São Paulo, Pirassununga 13635-900, SP, Brazil; 3 Department of Animal Production and Preventive Veterinary Medicine, School of Veterinary Medicine and Animal Science, São Paulo State University, Botucatu 18618-681, SP, Brazil

**Keywords:** acid protease, *Bacillus licheniformis*, enzymes, weaned piglets

## Abstract

The aim of this study was to evaluate two proteases (P1 and P2) on the apparent and standardized ileal digestibility (AID and SID, respectively), of crude protein (CP) and amino acids (AAs) and apparent total tract digestibility (ATTD) of nutrients and gross energy (GE) of corn and soybean meal, with 46% and 48% CP, in pigs by the index method. Ninety crossbred castrated male piglets (13.52 ± 1.96 kg body weight) were fed a nitrogen-free diet, or diets containing corn or soybean meal as the sole sources of CP and AAs, supplemented or not with 0.05% of P1 or 0.01% of P2, an acid protease. Treatment differences were considered statistically significant at *p* < 0.05. The inclusion of proteases in corn and of P1 in soybean meal diets did not improve the digestibility of nutrients and energy. Otherwise, the addition of P2, an acid protease, in soybean meal diets increased (*p* < 0.05) the AID and SID of Lys, Met, Phe, Ala, Cys, and Glu, and the respective digestible AA values, from 7.5% to 22%, compared to soybean meal without proteases. The use of the acid protease can be an important tool to enhance the digestibility of the AAs of soybean meal in piglets.

## Simple Summary

After weaning, young pigs do not produce adequate amounts of enzymes to digest the proteins from vegetable feed ingredients, which are major constituents of their diets. The addition of exogenous protease to pig diets may increase the digestion of dietary protein, improving the utilization of protein and amino acids from the animals’ diets. Thus, the objective of this study was to evaluate two different proteases (P1 and P2) on the digestibility of the protein and amino acids from corn and two sources of soybean meal, with 46% and 48% crude protein, in piglets (13.52 ± 1.96 kg body weight). The two proteases did not influence the digestion of corn protein and amino acids, and P1 did not affect the digestion of protein and amino acids of the two sources of soybean meal. The addition of P2, an acid protease, in soybean meal diets increased the digestion of leucine, lysine, methionine, phenylalanine, alanine, cystine, and glutamate, and their respective digestible values, from 7.5% to 22%, compared to soybean meal without proteases. Thus, P2 was effective in improving the utilization of several amino acids from soybean meal in piglets, enhancing the nutritional value of soybean meal for piglets.

## 1. Introduction

Weaning is a critical moment for piglets, as the animals are submitted simultaneously to immunological, environmental, social, and nutritional stress [1,2]. Specifically, the change from the sow’s milk, which is very nutritious, digestible, and palatable for piglets, to a dry diet, composed mainly of vegetable feed ingredients, which are less digestible and palatable than milk, is challenging for the piglets, and can damage the intestinal villi and disrupts intestinal health [3,4,5]. Piglets can lose from 100 to 250 g body weight on the first day post-weaning and require up to two weeks to recover to pre-weaning energy intake levels [5].

Corn and soybean meal are the most used feedstuffs in swine feeding, but are not properly digested by young pigs, due to the unsatisfactory synthesis of enzymes for degrading its chemical components [6]. Moreover, soybean meal, even after thermal processing, may contain antinutritional factors (ANFs) such as lectins, glycinin, β-conglycinin, and residual trypsin inhibitors [7,8,9,10]. These compounds can impair protein digestibility and, in the case of glycinin and β-conglycinin, may also trigger hypersensitivity reactions in piglets [8,10]. Therefore, significant undigested fractions of these vegetable feed ingredients will remain in the animals’ intestine and can serve as a substrate for harmful microorganisms. These dietary changes can compromise the intestinal health of piglets, cause diarrhea, and reduce growth performance [11].

There are some dietary strategies to increase the nutrient digestibility of vegetable feedstuff and to eliminate the detrimental effects of their ANFs in piglets, including the supplementation of exogenous enzymes to diets [6]. Exogenous enzymes are not produced by pigs, and their addition in pig feeding can be a tool to maximize the use of low-nutritional-value ingredients, avoiding enteric problems, reducing environmental issues, and production costs [12,13]. Among the available enzymes to be used in animal feeding, exogenous proteases have the potential to improve the digestibility of amino acids (AAs) and crude protein (CP) of feed ingredients, consequently reducing nitrogen excretion [14], assisting the action of endogenous enzymes, especially in newly weaned piglets that have insufficient production of digestive enzymes [15]. In addition, proteases may degrade the ANFs of protein origin, such as allergenic proteins and trypsin inhibitors [16].

In most studies, exogenous enzymes were supplied to piglets as enzyme cocktails, and the effects of specific proteases on monogastric animals were not clearly studied [17,18]. Another relevant factor is that the potential increase in AA digestibility derived from proteolytic enzymes must be tested and precisely quantified, allowing the creation of nutritional matrices of the feed enzymes [19,20,21]. In other words, it is essential to account for the surplus of nutrients resulting from the dietary addition of feed enzymes, allowing precise feed formulation, and enabling the nutritional valorization of feed ingredients. Such points would permit the reduction in the inclusion of crystalline AA or protein sources in diets, formulating them with lower CP contents [22,23].

Proteases differ in their optimal pH of activity, which determines the site and potential efficacy of their action in the gastrointestinal tract of pigs. Acid proteases act in the stomach at low pH, facilitating the early denaturation and hydrolysis of dietary proteins before the chyme reaches the small intestine, reducing the activity of protein ANFs in this site of the gastrointestinal tract [12,24,25]. In contrast, neutral or alkaline proteases act primarily in the small intestine, complementing the effects of pancreatic enzymes like trypsin and chymotrypsin [26].

The evaluation of alkaline and acid proteases will allow for the determination of which type of enzyme supplementation is more effective to improve the digestibility of nutrients in piglets. Additionally, it will be possible to elaborate the nutritional matrices of the enzymes, potentially improving the use of vegetable feed ingredients in piglets feeding. Thus, the aim of this study was to test the supplementation of two proteases, an alkaline and an acid source, on the apparent and standardized ileal digestibility (AID and SID, respectively), of AAs, CP, dry matter (DM), and on apparent total tract digestibility (ATTD) of CP, DM, and gross energy (GE) of corn and soybean meal, with 46% and 48% crude protein (SBM46 and SBM48, respectively), in young piglets.

## 2. Materials and Methods

This study was approved by the Ethics Committee on Animal Use of Luiz de Queiroz College of Agriculture (ESALQ), University of São Paulo (USP), protocol number 2018.5.1576.11.5. The trial was conducted at the research facilities of the Swine Production Sector of the Department of Animal Science of ESALQ-USP, located in Piracicaba, São Paulo, Brazil. The animals remained healthy during the experiment and no adverse events were observed.

A total of 90 castrated male pigs (13.52 ± 1.96 kg body weight (BW)), originating from the AGPIC 337 X Camborough crossbreed (Agroceres PIC, Patos de Minas, MG, Brazil), were allotted according to their initial BW in a randomized complete block design experiment, with 9 dietary treatments and a nitrogen-free (NF) diet, 9 repetitions per treatment, and 1 pig per experimental unit. The animals were housed individually in 3 m^2^ pens with partially slatted floors, equipped with a semi-automatic feeder, a nipple drinker, and an infrared lamp for heating. The ambient temperature was monitored with two Testo 174H NTC Dataloggers (Texto Inc.,Titisee-Neustadt, BW, Germany), distributed within the experimental facility.

The animals were fed one of ten diets (Table 1): a NF diet to determine the ileal endogenous losses of AAs and CP, composed mainly of corn starch, sugar, cellulose, and soybean oil; or diets that contained one of the test ingredients, corn, SBM46, and SBM48, as the sole sources of protein and AA, with or without protease 1 (P1) or protease 2 (P2). Corn was included at 95.74% and soybean meal at 30% in the test diets, replacing the cornstarch, sugar, cellulose, and soybean oil of NF diets. The P1 was obtained from *Bacillus licheniformis*, with an enzymatic activity of 600,000 u/g, and was included in the diets at 0.05%. The P2, which is an acid protease, had an enzymatic activity of 50,000 u/g and was added at 0.01% to the diets. Both enzymes were added to the diets according to the recommendations of the manufacturer, replacing the inert ingredient of the diets. Chromium oxide was included in all diets at 0.5% as an indigestible indicator. The diets were formulated to meet the nutritional requirements of minerals, vitamins and energy of the pigs [27].

The test feed ingredients, corn, SBM46, and SBM48, and the diets evaluated in this study were ground in a knife mill (model MA680, Marconi, Piracicaba, SP, Brazil) with a 1 mm sieve and analyzed for dry matter (DM; Method 934.01 [28]); gross energy (GE) in an adiabatic calorimeter (Model C5003 Control, IKA-Works, Wilmington, NC, USA); nitrogen (N; Method 990.03 [28]) to estimate crude protein (CP) content; neutral detergent insoluble fiber (NDF [29]) and acid detergent insoluble fiber (ADF [29]); ether extract (EE, Method 945.16 [28]); ash (Method 942.05 [28]); calcium (Ca; Method 968.08 [28]); phosphorus (P; Method 965.17 [28]); and AAs by high performance liquid chromatography (Method 982.30E [28]). The analyzed chemical composition of the test ingredients is in Table 2.

The experimental period lasted twelve days, seven days for the adaptation of animals to the diets and facilities, followed by four days for partial feces collection, and one day for ileal digesta collection, after the animals were euthanized. During feces collection, the animals were observed from 7 a.m. to 7 p.m. and feces were collected from the floor of the pens immediately after defecation, stored in plastic bags and frozen. Feces that had mixed with hair, urine, and feed leftovers were discarded. Ileal digesta collection was performed after the animals were euthanized, approximately 11 h after their last meal [30]. The animals were stunned by electronarcosis and sacrificed by exsanguination. Sequentially, a longitudinal ventral incision was made in the pigs to expose the digestive tract. The ileocecal junction was identified and from there towards the jejunum, a portion of approximately one linear meter was separated. All the digesta contained in this fraction of the ileum was collected and stored in a container containing formic acid solution (5%) and frozen.

At the end of the collection period, fecal samples were thawed, homogenized per experimental unit, subsampled, and dried at 55 °C in a forced air circulation oven (model MA035, Marconi, Piracicaba, SP, Brazil) for 72 h and were ground and analyzed for DM, GE, and total nitrogen, as previously described. The digesta samples were lyophilized (model LH 0401, Terroni, São Carlos, SP, Brazil), ground in an analytical mill (A11 Basic, IKA, Shanghai, China) to a fraction of 2 mm in diameter, and then subjected to DM and nitrogen determinations, as described for the other samples. The test feed ingredients and digesta samples were analyzed for AA content (method 994.12 [28]). Chromium quantifications were performed on diets, digesta, and feces samples after digestion in a solution containing nitric and perchloric acids, and hydrogen peroxide (4:2:2 *v*/*v*), under heating in a digestion block (model MA 850, Marconi, Piracicaba, SP, Brazil), and chromium readings were performed on an optical emission spectrophotometer with inductively coupled plasma (model Optima 8300, Perkin Elmer, Shelton, CT, USA).

The AID and SID of AAs and CP in all test diets, and the ATTD of GE and CP of corn diets were calculated by the direct method, as soybean meal and corn were the only source of AAs and CP in all test diets, and corn was the only source of GE in the corn diets. The ATTD of GE of the soybean meal samples was calculated by difference, considering the ATTD of GE in the NF diet and in the test diets. All calculations were performed according to the index approach [31].

Statistical analyses were performed using the R software (version 3. 7.1, R Core Team, Vienna, WI, Austria). The presence of outliers was assessed by checking if an observation had a studentized residual greater than 3 in an absolute value. The residual normality distribution was evaluated by the Cramer von–Misses test and the homogeneity of error variation was verified using the Hartley’s test.

The statistical evaluation of the AID, SID, and ATTD of nutrients and energy of corn and soybean meal were performed separately. The data from corn without enzymes, with P1, and with P2 were compared among each other, and the results from soybean meal treatments were evaluated in a 2 × 3 factorial design, considering the type of soybean meal (high and low protein) and the protease addition (without protease, P1, and P2). The data were subjected to analyses of variance, and the means were separated using Tukey’s test. Treatment differences were considered statistically significant at *p* < 0.05.

## 3. Results

The CP concentrations in corn, SBM46%, and SBM48% were 9.2%, 50.2%, and 55% (dry matter basis), respectively. Regarding the total AAs in corn, SBM46%, and SBM48%, the values were 9.58%, 47.81%, 52.51% (dry matter basis), respectively (Table 2).

The addition of P1 or P2 to the diets did not influence (*p* > 0.05) the AID and SID of CP and AAs, and the ATTD of DM, CP, and the GE of corn. Similarly, there was no effect (*p* > 0.05) of the enzymes on the nutrient and energy digestible values of corn (Table 3, Table 4, Table 5 and Table 6).

There was an interaction (*p* < 0.05) between the factors under study for the ATTD of DM and the GE of soybean meal (Table 5). The ATTD of DM and the GE of SBM48% without enzymes were greater (*p* < 0.05) than those of SBM48% with P1 and P2, which did not differ (*p* > 0.05), and that were greater (*p* < 0.05) than those observed in SBM46%, with or without proteases (*p* > 0.05).

Regarding the ingredient factor, it was found that the ATTD of CP, AID of Pro and Cys, and standardized ileal Pro of SBM48 were higher (*p* < 0.05) than those of SBM46. Similarly, except for Trp and Cys, the digestible, apparent, and standardized contents of all other AAs were higher (*p* < 0.05) in SBM48 than in SBM46 (Table 7).

The use of P2 increased (*p* < 0.05), from 7% to 22%, the AID and SID of Met, Phe, Lys, and Glu, the AID of Arg and the SID of Leu, Ala, and Cys in relation to soybean meal with P1 and without proteases. Additionally, the AID and SID of Ile, His, Asp, and Ser, the AID of Leu and the SID of Arg of the ingredients with P2 were higher than those verified with P1 but did not differ (*p* > 0.05) from those observed in the ingredients without the enzymes (Table 7 and Table 8).

The apparent and standardized ileal digestible contents of Met, Ile, Phe, Lys, Arg, Ser, Glu, Ala, Cys, and Tyr were higher (*p* < 0.05) in soybean meal with the addition of P2 than with P1 or without proteases. The use of P2 did not alter (*p* > 0.05) the digestible contents of Val, His, and Asp in relation to soybean meal without the enzymes; however, these values were higher (*p* < 0.05) than those verified in the ingredients that had the addition of P1 (Table 9 and Table 10).

## 4. Discussion

The present study evaluated the effect of supplementing two proteases (P1 and P2) on the AID and SID of CP and AAs, as well as on the ATTD of nutrients and energy in corn and soybean meal for newly weaned piglets. The results obtained indicate that the impact of the proteases depended on the ingredient tested and on the type of enzyme used.

Few studies have been conducted to evaluate the effects of exogenous proteases on the nutrient digestibility of corn. The initial expectation was that proteases could aid in the digestion of the protein matrix surrounding the starch granules [32] whose function is to contain them and increase the rigidity of the endosperm [12]. By degrading this matrix, the protease would increase the exposure of starch to amylase, thereby improving the digestibility of energy and protein. However, in the present study, no significant differences were observed in the nutrient digestibility of corn with the addition of any of the tested proteases.

The absence of an effect may be attributed to multiple factors. Studies have shown that, in corn-based diets, the response to protease supplementation may be limited. First, the protein content of corn is relatively low, which ranged from 9 to 9.3% of the grain’s dry matter in this study, and, possibly, the pigs’ endogenous enzymes were sufficient to degrade the proteins of the feed ingredient, making the addition of proteases unnecessary [33,34]. Moreover, the protein matrix of corn is composed mainly of zeins, which are hydrophobic and soluble only in alcoholic solvents, forming a barrier resistant to protease action and limiting the access of amylolytic enzymes to starch [35]. It is also possible that the proteases tested were not specific to corn proteins [35].

In addition, a study of growing and finishing pigs has also reported no effect of protease in predominantly corn-based diets [33]. In contrast, more recent work suggests that the combined use of proteases with other enzymes (e.g., xylanase and phytase) may release additional substrates and improve corn digestibility [34]. This suggests that the isolated action of protease is insufficient in low-protein ingredients, like corn, but that multi-enzyme strategies could be more effective.

The supplementation of soybean meal diets with protease P1, which exhibits higher activity at a neutral-to-alkaline pH, did not improve nutrient digestibility. This may be explained by the intense secretion of endogenous enzymes (trypsin and chymotrypsin) in the small intestine, where P1 acts, which may have turned P1 unnecessary or redundant. Additionally, the thermal processing of soybean meal may have led to the formation of protein aggregates resistant to hydrolysis by P1 [24,25,36]. These results indicate that the choice of protease should consider its pH of activity and the type of available substrate.

In contrast to the lack of improvement of the nutrient digestibility of corn, with P1 and P2 added, and of soybean meal with P1 supplementation, the inclusion of the acid protease P2 increased from 6 to 8% the AID and SID of most essential AAs of soybean meal diets, compared with the control diets. This result is consistent with the high content of CP in SBM46 and SBM48, providing a great amount of substrate for the enzymes, and possibly with the action of the acid protease at gastric pH. It can be speculated that the acid protease could have acted in the acid conditions of the stomach of the pigs, denaturing or digesting all the protein pool, including the proteinaceous ANFs from the soybean sources. Possibly, the ANFs of soybean meal were degraded in the stomach of the pigs before reaching the small intestine. Therefore, the possible negative effects of the ANFs, including potential reductions on the digestibility of nutrients, were avoided because of the action of P2 [21,24,25]. In other words, the acidic P2 may have had two important functions, aiding the digestion of the total CP per se and degrading the ANF of soybean before these compounds could reach the small intestine and cause any kind of problems to the pigs.

Previous studies have shown that protease supplementation in piglet diets containing soybean meal increased AA digestibility l [25] and improved DM and CP digestibility in weanling pigs [21,37]. Similar results were obtained in other studies, in which protease improved the ATTD of DM by 3.45% and reduced NH_3_ emissions in weanling pigs fed corn–soybean meal diets [38], and also the use of proteases could restore piglet performance to levels like those achieved with higher CP diets in reduced-protein diets [12,39]. But despite the benefits observed, the literature still presents inconsistent results. Some authors have reported no improvement in the digestibility of nutrients in soybean meal with proteases, especially when dietary CP already exhibited high digestibility [40,41]. Other studies have shown that solely alkaline proteases were not effective, but their effect was enhanced when combined with carbohydrases [42,43]. These discrepancies may be associated with differences in animal age, enzyme inclusion levels, soybean meal processing, and variations in the specific activity of the proteases used. Other factors that may contribute to enzymatic inefficiency include inactivation in the stomach by pepsin [44] or loss of efficacy during high-temperature feed processing. There are also reports that exogenous proteases could impair digestibility by reducing the production of pancreatic enzymes [45,46], an effect that was not measured in the present study.

There are other approaches used to reduce ANFs and increase the AID and SID of AAs and CP of soybean meal in piglets, such as different processing techniques of the feedstuff (fermentation, enzyme treatment, pelleting, and extrusion) or the use of refined soybean products, such as soy protein concentrates [7,8,9,47,48]. Studies with nursery pigs have shown that the effects of processing techniques on AA digestibility are variable, usually ranging from small improvements up to around 10%, while soy protein concentrate shows more consistent increases [8,47,48,49]. In the present study, supplementation with the acid protease P2 increased the digestibility of soybean meal AAs from 7.5 to 22%, which is within or above the range reported for processed soybean products. These results indicate that the use of protease in feed can be a practical and flexible alternative to processed soybean meals, but further studies directly comparing both strategies are still required.

From a nutritional standpoint, the improvements in CP and AA digestibility observed with P2 represent a significant gain, as it may allow for a reduction in CP levels in diet formulations without compromising the supply of digestible AAs. In commercial settings, this improvement can be translated into a reduced need for supplementation with crystalline AAs as well and lowering nitrogen excretion that has a direct impact on production costs and the environmental sustainability of swine production.

This study achieved results about the use of two different proteases, highlighting the important difference between acid and alkaline sources of protease, and also provided the nutritional matrices of two types of soybean meal when supplemented with proteolytic enzymes. These findings are relevant from an academic point of view but also from an applied practical standpoint. The data generated can be used for diet formulation when utilizing sources of soybean meal with 46 and 48% CP, supplemented with an acid protease. Future research is needed in order to evaluate other sources of proteases, in different concentrations, and to test other parameters such as growth performance, gut health and gut microbiota to fully elucidate the mechanisms of action and the potential of protease use in swine production.

## 5. Conclusions

The digestibility of corn was not influenced by the proteases used in the study, but the acid protease improved the AID and SID of AAs of soybean meal and can be an important tool to enhance the efficiency of use of AAs of soybean meal in piglets.

## Figures and Tables

**Table 1:** Ingredient and analyzed composition of experimental diets.

Iten	Diets									
	Corn			SBM46%			SBM48%			NF
	WE	P1	P2	WE	P1	P2	WE	P1	P2	
Ingredient, % (as-fed basis)										
Corn	95.74	95.74	95.74	-	-	-	-	-	-	-
Corn starch ^1^	-	-	-	46.38	46.38	46.38	46.38	46.38	46.38	67.07
Soybean meal 46%	-	-	-	30.00	30.00	30.00	-	-	-	-
Soybean meal 48%	-	-	-	-	-	-	30.00	30.00	30.00	-
Sugar ^2^	-	-	-	13.83	13.83	13.83	13.83	13.83	13.83	20.00
Cellulose ^3^	-	-	-	2.766	2.766	2.766	2.766	2.766	2.766	4.000
Soybean oil	-	-	-	2.766	2.766	2.766	2.766	2.766	2.766	4.000
Dicalcium phosphate	2.100	2.100	2.100	2.100	2.100	2.100	2.100	2.100	2.100	2.100
Salt	0.720	0.720	0.720	0.720	0.720	0.720	0.720	0.720	0.720	0.720
Limestone	0.600	0.600	0.600	0.600	0.600	0.600	0.600	0.600	0.600	0.600
Chromium oxide	0.500	0.500	0.500	0.500	0.500	0.500	0.500	0.500	0.500	0.500
Inert	0.050	-	0.040	0.050	-	0.040	0.050	-	0.040	-
Protease 1	-	0.050	-	-	0.050	-	-	0.050	-	-
Protease 2	-	-	0.010	-	-	0.010	-	-	0.010	-
Potassium carbonate, 98%	-	-	-	-	-	-	-	-	-	0.600
Magnesium oxide, 58%	-	-	-	-	-	-	-	-	-	0.120
Choline chloride, 60%	0.100	0.100	0.100	0.100	0.100	0.100	0.100	0.100	0.100	0.100
Trace mineral supplement ^4^	0.100	0.100	0.100	0.100	0.100	0.100	0.100	0.100	0.100	0.100
Vitamin supplement ^5^	0.090	0.090	0.090	0.090	0.090	0.090	0.090	0.090	0.090	0.090
Total	100.0	100.0	100.0	100.0	100.0	100.0	100.0	100.0	100.0	100.0
Analyzed composition, % or otherwise indicated (dry matter basis)										
Dry matter	89.2	88.6	89.9	91.0	91.3	91.1	91.3	91.7	91.7	91.4
Crude protein	9.00	9.20	9.30	15.8	15.8	13.6	16.0	18.0	15.9	0.82
Gross energy, kcal/kg	4294	4290	4282	4261	4236	4273	4307	4267	4254	4270
Ether extract	3.71	3.44	4.42	3.50	5.51	3.99	4.83	4.31	4.00	4.25
Neutral detergent fiber	8.90	8.90	8.00	6.60	7.40	6.20	5.28	5.90	5.44	3.21
Acid detergent fiber	1.91	2.26	1.64	4.26	4.31	3.64	3.00	3.38	3.05	1.64
Indispensable amino acids										
Arg	0.46	0.45	0.45	1.00	0.9	0.78	1.07	1.08	1.12	0.02
His	0.28	0.27	0.26	0.36	0.33	0.42	0.38	0.36	0.37	0.02
Ile	0.34	0.34	0.33	0.66	0.61	0.78	0.74	0.75	0.74	0.02
Leu	1.12	1.15	1.10	1.14	1.06	1.30	1.20	1.23	1.28	0.04
Lys	0.27	0.27	0.24	0.78	0.79	0.99	0.94	0.95	0.93	0.09
Met	0.21	0.18	0.18	0.21	0.19	0.25	0.23	0.24	0.26	0.02
Phe	0.45	0.43	0.41	0.68	0.66	0.80	0.77	0.77	0.8	0.04
Thr	0.36	0.35	0.34	0.59	0.56	0.68	0.65	0.65	0.59	0.00
Trp	0.04	0.03	0.03	0.18	0.11	0.10	0.16	0.15	0.13	0.03
Val	0.44	0.45	0.44	0.69	0.64	0.80	0.74	0.74	0.76	0.02
Dispensable amino acids										
Ala	0.65	0.64	0.64	0.63	0.57	0.74	0.66	0.66	0.77	0.02
Asp	0.57	0.52	0.46	1.52	1.31	1.76	1.72	1.56	1.71	0.03
Cys	0.18	0.15	0.12	0.22	0.20	0.31	0.28	0.32	0.4	0.10
Glu	1.67	1.59	1.56	2.41	2.18	2.85	2.60	2.63	2.51	0.04
Gly	0.35	0.33	0.33	0.58	0.54	0.68	0.61	0.61	0.67	0.00
Pro	0.87	0.81	0.81	0.70	0.64	0.82	0.74	0.75	0.83	0.02
Ser	0.43	0.39	0.39	0.66	0.59	0.76	0.68	0.69	0.74	0.00
Tyr	0.36	0.34	0.34	0.49	0.45	0.59	0.55	0.54	0.59	0.02
Total amino acids	9.00	8.67	8.39	13.3	12.2	15.7	15.6	14.5	15.1	0.54

**SBM46%**, soybean meal 46% CP; **SBM48%**, soybean meal 48% CP; **NF**, nitrogen free; **WE**, without enzyme; **P1**, protease 1; **P2**, protease 2. ^1^ Ingredion Brasil Ingredientes Industriais Ltd.a., Mogi Guaçu, SP, Brasil. ^2^ Açucareira Boa Vista Ltd.a., Limeira, SP, Brazil. ^3^ J. Rettenmaier & Söhne Corp., Rosenberg, OS, Germany. ^4^ Quantity per kg of feed: iron 100.00 mg/kg, manganese 60.00 mg/kg, zinc 119.00 mg/kg, cupper 15.00 mg/kg, iodine 1.50 mg/kg, and selenium 0.63 mg/kg. ^5^ Quantity per kg of feed: Vit. A 9.90 UI/g, Vit. D3 3,15 UI/g, Vit. E 45.00 mg/kg, Vit. K 5.40 mg/kg, Vit. B1 4.50 mg/kg, Vit. B2 11.70,mg/kg, Vit. B6 6.30 mg/kg, Vit. B12 54.00 mcg/kg, Vit. B3 63.00 mg/kg, Vit. B5 36.00 mg/kg, Vit. B9 1.26 mg/kg, and Vit. B7.

**Table 2:** Analyzed chemical composition of feed ingredients (%, or otherwise indicated, dry matter basis).

	Ingredients		
Item	Corn	Soybean Meal 46% CP	Soybean Meal 48% CP
Dry matter	88.39	89.06	89.51
Crude protein	9.2	50.2	55.0
Gross energy, kcal/kg	4574.29	4720.04	4746.83
Ether extract	6.11	3.12	2.83
Neutral detergent fiber	12.8	12.7	9.1
Acid detergent fiber	2.87	7.22	4.12
Indispensable amino acids			
Arg	0.09	0.73	0.74
His	0.37	2.00	2.13
Ile	1.30	3.87	4.18
Leu	0.27	3.23	3.51
Lys	0.49	2.59	2.76
Met	0.37	2.27	2.61
Phe	0.19	0.45	0.60
Thr	0.46	3.59	3.85
Trp	0.48	2.44	2.67
Val	0.26	1.27	1.44
Dispensable amino acids			
Ala	0.87	2.49	2.69
Asp	0.71	2.23	2.47
Cys	0.44	2.43	2.67
Glu	0.15	0.85	0.66
Gly	0.33	2.09	2.28
Pro	1.86	8.66	9.82
Ser	0.63	5.56	6.22
Tyr	0.38	1.79	1.93
Total amino acids	9.58	47.81	52.51

**Table 3:** Apparent total tract digestibility (%) (ATTD) of dry matter (DM), crude protein (CP), and gross energy (GE), and apparent ileal digestibility (%) (AID) of DM and amino acids (AAs) of corn, without enzymes or supplemented with two types of proteases, in piglets.

Item	Corn			SEM	*p* Value
	Without Enzyme	Protease 1	Protease 2		
ATTD					
DM	92.76	91.52	92.66	0.13	0.793
CP	83.37	84.25	85.50	0.67	0.444
GE	92.27	92.03	92.88	8.55	0.425
AID					
DM	74.44	76.81	79.36	1.42	0.374
Indispensable AA					
Arg	68.22	73.06	77.70	1.86	0.086
His	71.95	73.67	76.54	1.60	0.493
Ile	65.28	70.34	73.72	2.00	0.212
Leu	74.82	79.12	82.89	1.62	0.113
Lys	50.11	53.66	53.45	2.54	0.838
Met	76.33	70.00	82.13	1.65	0.116
Phe	91.26	73.85	76.58	1.72	0.448
Thr	56.23	59.67	61.26	2.18	0.637
Trp	39.51	22.74	39.98	5.03	0.353
Val	65.33	70.84	74.69	1.99	0.140
Dispensable AA					
Ala	70.31	74.18	77.33	1.74	0.249
Asp	64.08	69.59	67.03	2.48	0.697
Cys	60.57	53.97	59.10	2.76	0.626
Glu	75.97	78.79	83.40	1.54	0.124
Gly	33.24	39.15	41.80	3.46	0.596
Pro	64.22	66.82	69.12	1.82	0.548
Ser	60.70	62.92	69.20	1.97	0.179
Tyr	66.65	69.19	73.62	1.91	0.312

**SEM**, Standard error of the mean.

**Table 4:** Standardized ileal digestibility (%) (SID) of amino acids (AAs) and crude protein (CP) of corn without enzymes or supplemented with two types of proteases, in piglets.

Item	Corn			SEM	*p* Value
	Without Enzyme	Protease 1	Protease 2		
SID					
Indispensable AA					
Arg	76.18	81.17	85.83	1.87	0.080
His	76.59	78.48	72.58	1.61	0.438
Ile	71.49	76.51	80.12	2.01	0.199
Leu	78.52	82.72	86.66	1.62	0.109
Lys	62.67	57.99	67.75	2.67	0.329
Met	80.08	78.42	86.57	1.66	0.102
Phe	76.26	79.08	82.12	1.73	0.380
Thr	66.76	69.90	71.86	2.18	0.635
Trp	70.99	61.23	77.21	4.89	0.435
Val	71.50	76.82	80.84	1.99	0.142
Dispensable AA					
Ala	75.16	79.08	82.24	1.75	0.243
Asp	69.34	75.39	73.55	2.49	0.630
Cys	68.22	63.33	70.19	2.76	0.603
Glu	79.23	81.06	86.88	1.55	0.098
Gly	59.10	66.61	69.35	3.51	0.476
Pro	87.15	95.34	93.87	1.93	0.194
Ser	70.23	73.20	79.51	1.99	0.140
Tyr	73.21	76.14	80.60	1.93	0.277

**SEM**, Standard error of the mean.

**Table 5:** Apparent ileal digestible (%) amino acid (AA) contents of corn (dry matter basis), without enzymes or supplemented with two types of proteases, in young piglets.

Item	Corn			SEM	*p* Value
	Without Enzyme	Protease 1	Protease 2		
Indispensable AA					
Arg	0.316	0.339	0.360	0.009	0.086
His	0.187	0.162	0.199	0.004	0.493
Ile	0.244	0.263	0.275	0.007	0.212
Leu	0.973	1.029	1.078	0.021	0.113
Lys	0.136	0.146	0.145	0.007	0.838
Met	0.147	0.142	0.158	0.003	0.116
Phe	0.347	0.359	0.373	0.008	0.448
Thr	0.210	0.223	0.229	0.008	0.637
Trp	0.041	0.020	0.033	0.005	0.226
Val	0.310	0.337	0.355	0.009	0.140
Dispensable AA					
Ala	0.501	0.529	0.551	0.012	0.249
Asp	0.406	0.441	0.425	0.016	0.697
Cys	0.089	0.079	0.087	0.004	0.626
Glu	1.409	1.462	1.547	0.028	0.124
Gly	0.109	0.128	0.137	0.011	0.596
Pro	0.559	0.582	0.602	0.016	0.548
Ser	0.268	0.278	0.305	0.009	0.179
Tyr	0.256	0.266	0.283	0.007	0.312

**SEM**, Standard error of the mean.

**Table 6:** Standardized ileal digestible (%) amino acid (AA) contents of corn (dry matter basis), without enzymes or supplemented with two types of proteases, in young piglets.

Item	Corn			SEM	*p* Value
	Without Enzyme	Protease 1	Protease 2		
Indispensable AA					
Arg	0.353	0.376	0.39	0.009	0.080
His	0.199	0.204	0.212	0.004	0.438
Ile	0.267	0.286	0.299	0.007	0.199
Leu	1.022	1.076	1.127	0.021	0.109
Lys	0.170	0.157	0.164	0.007	0.329
Met	0.154	0.151	0.166	0.003	0.102
Phe	0.371	0.385	0.399	0.008	0.380
Thr	0.249	0.261	0.268	0.008	0.635
Trp	0.069	0.054	0.068	0.005	0.357
Val	0.340	0.365	0.384	0.009	0.142
Dispensable AA					
Ala	0.536	0.564	0.586	0.012	0.243
Asp	0.439	0.478	0.466	0.016	0.630
Cys	0.100	0.093	0.103	0.004	0.603
Glu	1.470	1.504	1.612	0.029	0.98
Gly	0.194	0.219	0.228	0.012	0.476
Pro	0.759	0.830	0.818	0.017	0.194
Ser	0.310	0.323	0.351	0.009	0.140
Tyr	0.282	0.293	0.310	0.007	0.277

**SEM**, Standard error of the mean.

**Table 7:** Apparent total tract digestibility (%) (ATTD) of dry matter (DM), crude protein (CP), and gross energy (GE), and apparent ileal digestibility (%) (AID) of DM and amino acids (AAs) of soybean meal with 46% and 48% CP, without enzyme (WE) or supplemented with two types of proteases (P1 and P2), in piglets.

Item	Soybean Meal 46% CP			Soybean Meal 48% CP			SEM	*p* Value		
	WE	P1	P2	WE	P1	P2		Ing	Enz	Ing × Enz
ATTD										
DM	93.43 ^c^	93.39 ^c^	93.58 ^c^	94.65 ^a^	93.95 ^b^	93.84 ^b^	0.09	<0.001	0.085	0.042
CP	91.31	91.55	91.00	92.97	92.89	91.83	0.18	<0.001	0.063	0.526
GE	92.94 ^c^	90.44 ^c^	93.63 ^c^	95.07 ^a^	95.07 ^b^	94.10 ^b^	6.57	<0.001	0.056	0.005
AID										
DM	83.01	79.44	82.39	83.46	81.69	81.69	0.83	0.430	0.358	0.859
Indispensable AA										
Arg	83.39 ^b^	80.87 ^b^	89.87 ^a^	84.94 ^b^	84.00 ^b^	91.23 ^a^	1.13	0.551	0.012	0.942
His	79.29 ^ab^	73.14 ^b^	86.34 ^a^	80.70 ^ab^	79.54 ^b^	85.51 ^a^	1.32	0.576	0.021	0.535
Ile	81.13 ^ab^	75.60 ^b^	86.18 ^a^	82.35 ^ab^	82.55 ^b^	87.74 ^a^	1.13	0.300	0.023	0.527
Leu	80.08 ^ab^	74.27 ^b^	84.25 ^a^	80.22 ^ab^	81.06 ^b^	86.68 ^a^	1.16	0.345	0.030	0.504
Lys	71.83 ^b^	71.30 ^ab^	84.36 ^a^	76.96 ^b^	79.40 ^ab^	86.36 ^a^	1.69	0.183	0.012	0.757
Met	82.32 ^b^	78.51 ^b^	89.25 ^a^	84.95 ^b^	85.80 ^b^	92.34 ^a^	1.10	0.088	0.002	0.610
Phe	80.39 ^b^	76.14 ^b^	87.05 ^a^	81.89 ^b^	82.44 ^b^	88.21 ^a^	1.12	0.286	0.008	0.578
Thr	71.85	64.50	81.02	75.00	76.76	73.45	1.66	0.647	0.356	0.065
Trp	84.04	64.79	77.10	73.90	74.85	74.85	1.89	0.823	0.431	0.205
Val	78.33 ^ab^	71.40 ^b^	84.02 ^a^	78.50 ^ab^	78.85 ^b^	84.68 ^a^	1.33	0.546	0.027	0.485
Dispensable AA										
Ala	74.21 ^b^	66.32 ^b^	83.81 ^a^	74.56 ^b^	76.73 ^b^	83.41 ^a^	1.53	0.420	0.004	0.266
Asp	78.25 ^ab^	73.48 ^b^	86.71 ^a^	82.16 ^ab^	80.37 ^b^	87.11 ^a^	1.35	0.301	0.011	0.608
Cys	65.96 ^b^	61.93 ^b^	83.94 ^a^	74.89 ^b^	77.49 ^b^	87.03 ^a^	1.90	0.019	<0.001	0.298
Glu	78.53 ^b^	79.73 ^ab^	87.82 ^a^	81.75 ^b^	83.81 ^ab^	89.24 ^a^	1.33	0.365	0.021	0.919
Gly	50.80	22.85	57.01	54.13	63.39	61.72	3.26	0.075	0.193	0.059
Pro	69.22	59.82	70.28	74.80	72.67	84.67	1.94	0.015	0.090	0.533
Ser	72.49 ^ab^	63.28 ^b^	83.81 ^a^	74.66 ^ab^	76.58 ^b^	79.40 ^a^	1.68	0.362	0.024	0.093
Tyr	78.58 ^ab^	73.81 ^b^	84.56 ^a^	82.10 ^ab^	81.71 ^b^	85.93 ^a^	1.10	0.115	0.028	0.481

**WE**, without enzyme; **P1**, protease 1; **P2**, protease 2; **SEM**, standard error of the mean; **Ing**, ingredient; **Enz**, enzyme. Values within a row with different superscripts differ by Tukey test at *p* < 0.05.

**Table 8:** Standardized ileal digestibility (%) (SID) of amino acids (AAs) of soybean meal with 46% and 48% crude protein, without enzyme (WE) or supplemented with two types of proteases (P1 and P2), in piglets.

Item	Soybean Meal 46% CP			Soybean Meal 48% CP			SEM	*p* Value		
	WE	P1	P2	WE	P1	P2		Ing	Enz	Ing × Enz
SID										
Indispensable AA										
Arg	87.05 ^ab^	84.94 ^b^	93.08 ^a^	88.35 ^ab^	87.39 ^b^	94.48 ^a^	1.12	0.624	0.019	0.974
His	82.88 ^ab^	77.10 ^b^	89.46 ^a^	84.10 ^ab^	83.16 ^b^	89.02 ^a^	1.30	0.576	0.028	0.595
Ile	84.29 ^ab^	79.00 ^b^	88.86 ^a^	85.15 ^ab^	85.33 ^b^	90.55 ^a^	1.12	0.351	0.031	0.581
Leu	83.71 ^b^	78.18 ^b^	89.34 ^a^	83.67 ^b^	86.75 ^b^	89.93 ^a^	1.15	0.384	0.020	0.542
Lys	76.17 ^b^	75.58 ^ab^	87.78 ^a^	80.55 ^b^	82.97 ^ab^	90.01 ^a^	1.67	0.226	0.016	0.820
Met	86.15 ^b^	82.81 ^b^	92.42 ^a^	88.43 ^b^	89.14 ^b^	95.29 ^a^	1.07	0.128	0.005	0.701
Phe	83.69 ^b^	79.55 ^b^	89.85 ^a^	84.82 ^b^	85.34 ^b^	91.02 ^a^	1.11	0.345	0.012	0.621
Thr	77.88	70.90	86.28	80.53	82.23	79.53	1.64	0.676	0.382	0.102
Trp	90.73	75.82	89.10	81.39	83.07	83.79	1.90	0.345	0.398	0.295
Val	82.23 ^ab^	76.65 ^b^	87.39 ^a^	82.13 ^ab^	82.50 ^b^	88.22 ^a^	1.31	0.590	0.039	0.534
Dispensable AA										
Ala	79.24 ^b^	71.85 ^b^	88.10 ^a^	79.36 ^b^	81.47 ^b^	87.47 ^a^	1.50	0.494	0.009	0.304
Asp	80.24 ^ab^	75.76 ^b^	88.42 ^a^	83.91 ^ab^	82.30 ^b^	88.87 ^a^	1.34	0.325	0.015	0.646
Cys	72.21 ^b^	68.90 ^b^	88.41 ^a^	79.71 ^b^	81.83 ^b^	90.43 ^a^	1.80	0.057	<0.001	0.391
Glu	80.80 ^b^	82.23 ^ab^	89.73 ^a^	83.85 ^b^	85.88 ^ab^	91.42 ^a^	1.32	0.378	0.024	0.954
Gly	66.24	39.59	70.22	68.78	78.11	75.23	3.20	0.086	0.312	0.075
Pro	97.74	91.39	94.62	101.7	99.33	108.8	1.82	0.036	0.439	0.476
Ser	78.65 ^ab^	70.15 ^b^	89.17 ^a^	80.64 ^ab^	82.49 ^b^	84.87 ^a^	1.64	0.411	0.042	0.121
Tyr	83.35	78.82	88.53	86.40	86.03	89.93	1.07	0.150	0.051	0.554

**WE**, without enzyme; **P1**, protease 1; **P2**, protease 2; **SEM**, standard error of the mean; **Ing**, ingredient; **Enz**, enzyme. Values within a row with different superscripts differ by Tukey test at *p* < 0.05.

**Table 9:** Apparent ileal digestible (%) amino acid (AA) contents of soybean meal with 46% and 48% crude protein (dry matter basis), without enzyme (WE) or supplemented with two types of proteases (P1 and P2), in piglets.

Item	Soybean Meal 46% CP			Soybean Meal 48% CP			SEM	*p* Value		
	WE	P1	P2	WE	P1	WE		Ing	Enz	Ing × Enz
Indispensable AA										
Arg	2.300 ^b^	2.955 ^ab^	3.229 ^a^	3.274 ^b^	3.426 ^ab^	3.558 ^a^	0.046	<0.001	0.013	0.589
His	1.037 ^ab^	0.928 ^b^	1.096 ^a^	1.197 ^ab^	1.191 ^b^	1.279 ^a^	0.021	<0.001	0.003	0.278
Ile	1.840 ^b^	1.715 ^b^	1.998 ^a^	2.153 ^b^	2.158 ^b^	2.294 ^a^	0.037	<0.001	0.009	0.487
Leu	3.102 ^b^	2.877 ^b^	3.337 ^a^	3.352 ^b^	3.387 ^b^	3.621 ^a^	0.052	<0.001	0.011	0.468
Lys	2.323 ^b^	2.306 ^ab^	2.728 ^a^	2.700 ^b^	2.785 ^ab^	3.030 ^a^	0.062	0.002	0.013	0.813
Met	0.370 ^b^	0.353 ^ab^	0.401 ^a^	0.513 ^b^	0.518 ^ab^	0.557 ^a^	0.012	<0.001	0.002	0.677
Phe	2.085 ^b^	1.975 ^b^	2.258 ^a^	2.260 ^b^	2.275 ^b^	2.434 ^a^	0.033	0.001	0.009	0.621
Thr	1.436	1.289	1.619	1.600	1.638	1.567	0.036	0.057	0.397	0.075
Trp	0.615	0.474	0.564	0.550	0.557	0.557	0.014	0.892	0.441	0.213
Val	1.908 ^ab^	1.740 ^b^	2.099 ^a^	2.096 ^ab^	2.105 ^b^	2.261 ^a^	0.037	0.002	0.012	0.424
Dispensable AA										
Ala	1.658 ^b^	1.482 ^b^	1.873 ^a^	1.841 ^b^	1.894 ^b^	2.059 ^a^	0.040	<0.001	0.005	0.324
Asp	4.498 ^ab^	4.084 ^b^	4.819 ^a^	5.113 ^ab^	5.001 ^b^	5.420 ^a^	0.88	<0.001	0.012	0.631
Cys	0.563 ^b^	0.529 ^b^	0.716 ^a^	0.494 ^b^	0.511 ^b^	0.574 ^a^	0.016	<0.001	<0.001	0.120
Glu	6.799 ^b^	6.902 ^ab^	7.602 ^a^	8.028 ^b^	8.229 ^ab^	8.763 ^a^	0.151	<0.001	0.024	0.966
Gly	1.061	0.448	1.191	1.234	1.445	1.407	0.073	0.012	0.216	0.065
Pro	1.725	1.491	1.752	2.014	1.956	2.279	0.055	<0.001	0.081	0.508
Ser	1.758 ^b^	1.535 ^b^	1.997 ^a^	1.993 ^b^	2.045 ^b^	2.191 ^a^	0.046	<0.001	0.010	0.234
Tyr	1.358 ^b^	1.220 ^b^	1.510 ^a^	1.539 ^b^	1.528 ^b^	1.669 ^a^	0.030	<0.001	0.006	0.551

**WE**, without enzyme; **P1**, protease 1; **P2**, protease 2; **SEM**, standard error of the mean; **Ing**, ingredient; **Enz**, enzyme. Values within a row with different superscripts differ by Tukey test at *p* < 0.05.

**Table 10:** Standardized ileal digestible (%) amino acid (AA) contents of soybean meal with 46% and 48% crude protein (dry matter basis), without enzyme (WE) or supplemented with two types of proteases (P1 and P2), in piglets.

Item	Soybean Meal 46% CP			Soybean Meal 48% CP			SEM	*p* Value		
	WE	P1	P2	WE	P1	P2		Ing	Enz	Ing × Enz
Indispensable AA										
Arg	3.128 ^b^	3.101 ^ab^	3.344 ^a^	3.405 ^b^	3.556 ^ab^	3.684 ^a^	0.046	<0.001	0.019	0.642
His	1.082 ^ab^	0.978 ^b^	1.135 ^a^	1.246 ^ab^	1.243 ^b^	1.330 ^a^	0.021	<0.001	0.004	0.299
Ile	1.912 ^b^	1.836 ^ab^	2.059 ^a^	2.226 ^b^	2.289 ^ab^	2.367 ^a^	0.036	<0.001	0.027	0.470
Leu	3.243 ^b^	3.029 ^b^	3.461 ^a^	3.496 ^b^	3.528 ^b^	3.827 ^a^	0.052	<0.001	0.006	0.520
Lys	2.463 ^b^	2.444 ^ab^	2.839 ^a^	2.825 ^b^	2.910 ^ab^	3.157 ^a^	0.061	0.002	0.017	0.863
Met	0.387 ^b^	0.372 ^ab^	0.415 ^a^	0.533 ^b^	0.538 ^ab^	0.575 ^a^	0.012	<0.001	0.005	0.727
Phe	2.171 ^b^	2.063 ^b^	2.330 ^a^	2.340 ^b^	2.355 ^b^	2.512 ^a^	0.033	0.001	0.013	0.659
Thr	1.557	1.417	1.724	1.718	1.755	1.697	0.035	0.048	0.421	0.115
Trp	0.663	0.554	0.652	0.606	0.619	0.624	0.014	0.588	0.407	0.304
Val	2.004 ^ab^	1.843 ^b^	2.181 ^a^	2.193 ^ab^	2.203 ^b^	2.355 ^a^	0.037	0.002	0.017	0.477
Dispensable AA										
Ala	1.771 ^b^	1.605 ^b^	1.968 ^a^	1.959 ^b^	2.011 ^b^	2.160 ^a^	0.039	<0.001	0.010	0.363
Asp	4.608 ^ab^	4.211 ^b^	4.915 ^a^	5.221 ^ab^	5.121 ^b^	5.530 ^a^	0.088	<0.001	0.016	0.656
Cys	0.616 ^b^	0.588 ^b^	0.754 ^a^	0.525 ^b^	0.539 ^b^	0.596 ^a^	0.016	<0.001	<0.001	0.186
Glu	6.995 ^b^	7.119 ^ab^	7.768 ^a^	8.234 ^b^	8.433 ^ab^	8.977 ^a^	0.152	<0.001	0.027	0.986
Gly	1.383	0.827	1.466	1.567	1.780	1.714	0.072	0.009	0.343	0.082
Pro	2.436	2.278	2.358	2.739	2.674	2.930	0.054	<0.001	0.416	0.451
Ser	1.908 ^b^	1.701 ^b^	2.163 ^a^	2.153 ^b^	2.202 ^b^	2.337 ^a^	0.047	<0.001	0.014	0.237
Tyr	1.443 ^b^	1.314 ^b^	1.609 ^a^	1.622 ^b^	1.612 ^b^	1.766 ^a^	0.030	<0.001	0.006	0.536

**WE**, without enzyme; **P1**, protease 1; **P2**, protease 2; **SEM**, standard error of the mean; **Ing**, ingredient; **Enz**, enzyme. Values within a row with different superscripts differ by Tukey test at *p* < 0.05.

## Data Availability

The data supporting reported results can be provided at the request to the corresponding author.

## Abbreviations

The following abbreviations are used in this manuscript:

AA	Amino acids
AID	Apparent ileal digestibility
ANFs	Anti nutritional factors
ATTD	Apparent total tract digestibility
CP	Crude protein
DM	Dry matter
GE	Gross energy
NF	Nitrogen free
P1	Protease 1
P2	Protease 2
SBM46	Soybean meal with 46% crude protein content
SBM48	Soybean meal with 48% crude protein content
SID	Standardized ileal digestibility
WE	Without enzyme
